# Recent Studies and Prospects of Biologics in Allergic Rhinitis Treatment

**DOI:** 10.3390/ijms26104509

**Published:** 2025-05-09

**Authors:** Xiangning Cheng, Yue Zhou, Yuzhe Hao, Ziyi Long, Qianxue Hu, Bingyue Huo, Tianjian Xie, Shan Chen, Liuqing Zhou, Tao Zhou, Liyue Li, Qing Cheng, Jianjun Chen

**Affiliations:** Department of Otorhinolaryngology, Union Hospital, Tongji Medical College, Huazhong University of Science and Technology, Wuhan 430022, China

**Keywords:** allergic rhinitis, biologics, therapy

## Abstract

Allergic rhinitis (AR) is a common and increasingly prevalent chronic inflammatory disorder of the nasal mucosa that severely impacts patients’ quality of life, causing symptoms like nasal congestion, sneezing, and itching. AR is primarily mediated by immunoglobulin E (IgE) when allergens are present, making it challenging to manage despite available therapies like pharmacotherapy, immunotherapy, and surgery. Recently, research has focused on biologics as an emerging therapeutic option for AR. Biologics target specific immune pathways in type 2 inflammation, which underlies many allergic diseases including AR. Biologics offer a targeted and potentially more effective alternative to traditional therapies, addressing the underlying immune mechanisms rather than simply alleviating symptoms. Based on key clinical trial evidence, this paper tentatively proposes a multidimensional strategy for selecting biologics in AR, integrating serum IgE levels, disease phenotypes (seasonal/persistent), and comorbid characteristics to guide individualized treatment. However, the long-term cost-effectiveness, optimal dosing regimens, and patient adherence to biologics require further validation through real-world data. Despite these challenges, recent advancements in biologics represent a promising step forward in AR management. With ongoing research and clinical trials, biologics may soon provide more effective and lasting relief for patients suffering from allergic rhinitis.

## 1. Introduction

Allergic rhinitis (AR) is a type I hypersensitivity reaction mediated by immunoglobulin E (IgE) in individuals with an allergic predisposition following allergen exposure, clinically presenting with pruritus, sneezing, rhinorrhea, and nasal congestion [1]. AR affects nearly 400 million people worldwide, with incidence rates steadily rising annually [1,2]. Beyond the significant impact on quality of life from recurrent symptoms, AR also markedly increases the risk of developing other allergic conditions, including asthma, atopic dermatitis, sinusitis, and ocular conjunctivitis [3,4,5]. As such, effective therapeutic strategies aimed at achieving long-term symptom control and improving patient quality of life remain a critical focus in AR research.

According to established treatment guidelines [6], AR management strategies include allergen avoidance, pharmacological interventions, and immunotherapy. Pharmacological treatments primarily consist of first-line medications, such as nasal corticosteroids, second-generation oral and nasal antihistamines, and oral leukotriene receptor antagonists. Second-line medications include oral corticosteroids, oral and nasal mast cell stabilizers, nasal decongestants, and nasal anticholinergics [6]. While regular pharmacotherapy effectively manages mild-to-moderate AR symptoms, a subset of patients with severe AR does not respond adequately to standard medications [3].

Recent advancements in biologic therapies targeting type 2 immune responses have introduced promising new approaches for AR treatment. Omalizumab, a targeted IgE antibody, has shown significant therapeutic benefit for AR. A randomized controlled trial demonstrated that a single injection of 300 mg omalizumab administered two weeks before the pollen season onset more effectively alleviated symptoms and improved quality of life (QoL) scores for patients with seasonal allergic rhinitis (SAR) compared to standard pharmacotherapy [7]. This approach also reduces medication dependency, lessening the burden of prolonged treatment during the pollen season for SAR patients [7]. Additionally, biologics targeting IL-4/IL-13 pathways, exemplified by dupilumab, have shown favorable outcomes in AR treatment.

Despite the potential of biologic therapy, substantial gaps remain in understanding the full implications of biologics for AR. This review provides a comprehensive overview of recent advancements in biologic treatments for AR, and based on key clinical trial evidence, tentatively proposes a multidimensional strategy for selecting biologics that integrate serum IgE levels, disease phenotypes (seasonal/persistent), and comorbid characteristics to guide individualized AR treatment.

## 2. AR and Related Biologics

### 2.1. Allergic Rhinitis and Its Therapeutic Predicaments

Allergic rhinitis (AR), a prevalent immune-mediated chronic disorder, has witnessed a steady rise in its global incidence over recent years. This has not only significantly impaired patients’ quality of life but also emerged as a notable public health concern [8].

Multiple US-based surveys [9,10,11] have indicated that compared to individuals without AR, patients with AR commonly exhibit inferior sleep quality, heightened susceptibility to daytime drowsiness, and difficulties in maintaining concentration. Approximately 30% of these patients experience cognitive and memory impairments, and roughly 30% suffer from anxiety or depression. From the perspective of social productivity, 82% of adult patients encounter a decline in work quality, and 92% of pediatric patients experience a setback in academic performance. Moreover, AR imposes a substantial economic burden on patients, encompassing both direct medical expenses and indirect losses resulting from absenteeism. Inadequate symptom control or adverse drug reactions further exacerbate these consequences [12].

According to guidelines, current AR treatments mainly consist of allergen avoidance, drug therapy, and immunotherapy, with the specific plan determined by symptom severity and rhinitis type. For mild intermittent or mild persistent AR, second-generation H1 antihistamines or intranasal antihistamines are the first-line treatment options, relieving symptoms such as itching and sneezing by competitively blocking histamine H1 receptors. For moderate-to-severe persistent AR, intranasal corticosteroids, which suppress nasal mucosa inflammatory responses, are preferred. They can be used alone or in combination with other drugs but carry side-effect risks: the epistaxis incidence is 4–8% during short-term (2–12 weeks) use and rises to 20–28% after one year of use [12]. Intranasal antihistamines are well-tolerated but may cause nasal mucosa over-dryness with epistaxis and, in some cases, headaches [13]. However, many patients’ symptoms remain inadequately controlled, with only 32.7% of those receiving standard care (SoC) believing their symptoms are fully controlled [14].

AR treatment not only faces challenges in treatment efficacy and drug side-effects but also has a low patient compliance problem [13,14,15]. A survey in 13 major Chinese cities showed that 37.7% of patients did not follow the ARIA guideline-based standardized treatment, and 46.6% only took medicine during symptom flare ups [14]. The MASK study, using the Allergy Diary app for evaluation, found that in reality, only 11.3% of AR patients strictly adhered to the prescription (medication possession ratio ≥ 70%, proportion of covered days ≤ 1.25) [13]. Allergen-specific immunotherapy (SLIT), relying on patient self-management, has a relatively low overall compliance rate due to factors like insufficient awareness, difficulty in accessing specialized care, reimbursement policy limitations, long treatment courses, and safety concerns [16].

In summary, AR exerts a negative impact on patients in multiple aspects. Although existing treatments can control some symptoms, low compliance and suboptimal disease control persist. Biologics bring new hope for AR treatment. For instance, for patients with SAR, a single 300 mg injection of omalizumab two weeks before the pollen season can effectively relieve allergic symptoms during that time [7]. Compared with traditional multiple daily dose medications, it significantly reduces the patient’s medication burden and is expected to improve treatment compliance. For patients with refractory AR who do not respond to optimal drug treatment, biologics targeting the AR pathogenesis have shown promising application prospects in research and practice [17].

### 2.2. AR Inflammatory Pathway Spectrum Targeted by Biologics

**Figure 1:**
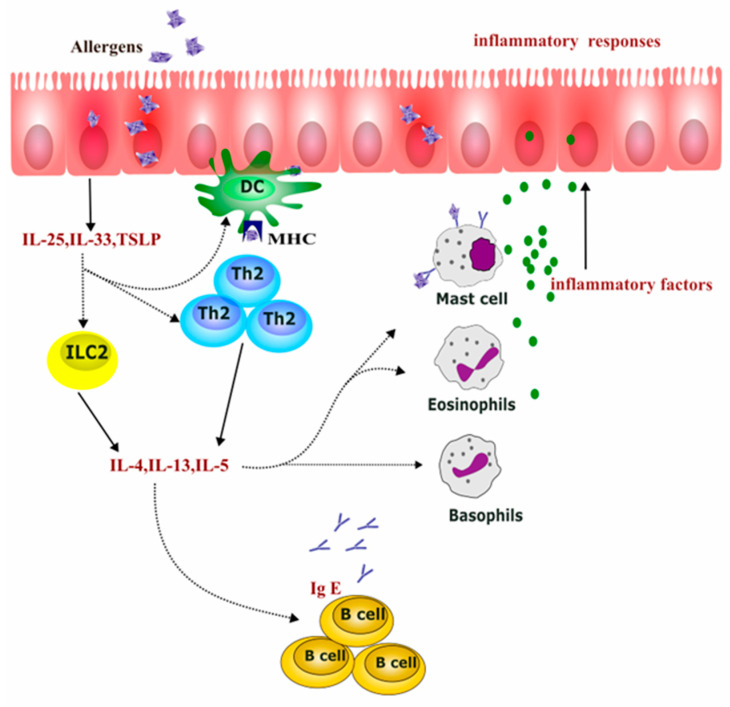
Type 2 inflammation in allergic rhinitis. After the invasion of allergens, dendritic cells (DCs) uptake and present antigens to drive Th2 cells. Damaged nasal epithelial cells produce pro-allergic cytokines IL-33, IL–25, and TSLP, which activate ILC2 and strengthen the responses of DCs and Th2 cells. The activated ILC2 and Th2 cells generate IL-4, IL-13, and IL-15, promoting IgE class switching, as well as the infiltration of mast cells, eosinophils, basophils and the release of inflammatory mediators, enhancing the local inflammatory response.

The type 2 immune response is characterized by type 2 cells such as Th2 cells and group 2 innate lymphoid cells (ILC2s), along with type 2 cytokines (e.g., IL-4, IL-5, IL-13, IL-25, IL-31, IL-33, and TSLP) [18,19,20]. Rapid advancements in immunology have highlighted type 2 immune-related inflammation as a critical pathogenic mechanism in allergic diseases, including AR [21,22]. When allergens invade the mucosal epithelium of AR patients, activated dendritic cells (DCs) present allergen peptides, promoting Th2 cell differentiation. Concurrently, damaged epithelial cells release epithelium-derived cytokines, such as thymic stromal lymphopoietin (TSLP), IL-25, and IL-33, which activate ILC2s, Th2 cells, and DCs [23,24,25]. The activation of type 2 immune cells leads to the release of various pro-inflammatory mediators, including IL-4, IL-5, and IL-13 [23,24]. These cytokines activate inflammatory cells, including mast cells, basophils, and eosinophils, and cause their accumulation in the nasal mucosa, leading to the release of a large number of inflammatory mediators, which is a key feature of chronic AR [26]. In addition, cytokines such as IL-4 and IL-13 are crucial for maintaining the Th2 response and stimulating IgE class switching [27,28]. Once IgE is produced, it binds to the high affinity functional immunoglobulin E receptors (FcεRI) on the surface of mast cells and basophils, a process known as sensitization [29]. Upon second and subsequent exposures to the same allergen, it binds to IgE on these sensitized cells, causing these cells to degranulate and release pre-formed and newly formed inflammatory mediators, such as prostaglandin D2, histamine, and leukotrienes. These mediators interact with nasal sensory nerves, blood vessels, and glands, thereby triggering the typical symptoms of AR [30,31,32,33,34,35,36] (see Figure 1).

Consequently, AR is often classified as a type 2 inflammatory disease [31,37]. Biologics targeting the type 2 immune response are being rapidly developed and have shown remarkable efficacy in treating type 2 inflammatory diseases, such as asthma, chronic sinusitis with nasal polyps, and atopic dermatitis [20,38,39,40]. Despite the overall progress in the field of type 2 immunology based biologics, developing biologics specifically for AR remains challenging. Given the similarities in the underlying type 2 immune-mediated pathophysiology, it is reasonable to hypothesize that these biologics may also hold therapeutic potential for AR. Biologics that target type 2 immunity (see Table 1) present significant opportunities for the effective management of AR. Their ability to precisely modulate the type 2 immune pathway makes them a promising approach for alleviating the symptoms and improving the quality of life for AR patients.

## 3. Research Progress and Classification of AR-Related Biologics

### 3.1. Anti-IgE

#### 3.1.1. Omalizumab

Omalizumab is an anti-IgE monoclonal antibody that reduces downstream inflammatory factors and blocks the inflammatory cascade triggered by allergen exposure. Its efficacy in treating allergic rhinitis has been well-documented in numerous studies [7,43,44,45]. For instance, a clinical trial by Kimihiro and colleagues found that a three-month course of subcutaneous omalizumab injections, administered every two or four weeks, significantly improved clinical symptoms in patients with moderate to severe SAR caused by Japanese cedar pollen [46]. Additionally, a meta-analysis of 16 studies involving 3458 patients confirmed that the omalizumab group demonstrated superior clinical scores, reduced emergency medication use, and improved quality-of-life scores compared to the control group, with no statistically significant differences in safety indicators [45]. In a prospective, randomized, controlled clinical trial in China, adding omalizumab to a guideline-based seasonal treatment regimen for fall SAR significantly reduced allergic symptoms, particularly ocular symptoms, and decreased the use of allergy medications when administered two weeks before the fall pollen season [7]. This resulted in a notable improvement in patients’ quality of life. Further real-world data from China showed that the total RQLQ score of 60 SAR patients treated with omalizumab improved from −2.55 (±1.04) to 0.47 (±0.47), representing a 2.08 (±1.01) point reduction (paired *t*-test, *p* < 0.001). The total nasal symptom score (TNSS) also decreased by 7.33 (±2.50) points (paired *t*-test, *p* < 0.001), supporting omalizumab’s effectiveness in real-world settings [43]. Omalizumab was approved for pollen-induced SAR in Japan in December 2019 [47]. Although it remains in the trial stage in China, treatment guidelines have recognized omalizumab as a viable option for managing allergic rhinitis.

#### 3.1.2. LP-003

LongBio Pharma announced preliminary Phase II data for LP-003, a next-generation anti-IgE antibody, at the 2024 American Academy of Allergy, Asthma & Immunology (AAAAI) annual meeting [48]. Results from its randomized controlled trial showed that patients with moderate-to-severe seasonal allergic rhinitis who received 100 mg LP-003 injections had significantly lower mean peak pollen nasal symptom scores (TNSS PPP) compared to the placebo group. Additionally, LP-003 is under investigation for potential use in treating chronic spontaneous urticaria, according to the Drug Clinical Trial Registration and Information Publication Platform. However, adverse events, including hyperuricemia, upper respiratory tract infections, and nasopharyngitis, were reported in more than 2% of treated patients.

### 3.2. Anti-IL4/IL13

#### 3.2.1. Dupilumab

Dupilumab is a human monoclonal IgG4 antibody that binds to the α-subunit of the IL-4 receptor, thereby blocking the signaling pathway shared by IL-4 and IL-13. In a study involving patients with asthma and perennial allergic rhinitis, dupilumab significantly improved the SNOT-22 total score (Sinusitis Nasal Outcome Test) and alleviated AR-related symptoms, such as nasal congestion, runny nose, and sneezing, compared to placebo [49]. Transcriptomic analysis indicated that 16 weeks of dupilumab treatment improved disease characteristics of AR, an effect not observed with subcutaneous immunotherapy (SCIT) alone [50]. Although the combination of SCIT and dupilumab did not significantly improve TNSS AUC (0–1 h) following nasal allergenic challenge (NAC) compared to SCIT alone, it offered potential advantages, such as improved SCIT tolerability and reduced risk of emergency medication use [51].

#### 3.2.2. CM310 (Spectrobab)

Developed by KEYMED BIOSCIENCES, CM310 is a monoclonal antibody targeting the human interleukin-4 receptor alpha subunit (IL-4R α). As the world’s first IL-4Rα antagonist approved for SAR, it has obtained approvals in China for treating SAR, atopic dermatitis, and chronic rhinosinusitis with nasal polyps [52,53]. Phase II and III clinical trials for CM310 in allergic rhinitis have been completed [41,54,55]. In a Phase II trial for the treatment of refractory seasonal allergic rhinitis (moderate to severe symptoms despite standard care), nasal symptoms significantly improved after 4 weeks of CM310 treatment [55]. Notably, patients with baseline blood eosinophils ≥300 cells/μL showed outstanding efficacy, and the drug exhibited good safety and tolerability [55]. The Phase III trial confirmed that a subcutaneous injection regimen every two weeks (q2w) significantly improved nasal and ocular symptoms, as well as quality of life with significant advantages in both the duration and proportion of symptom relief within 2–4 weeks [54]. These findings highlight CM310 as a novel treatment option for moderate-to-severe SAR. Its efficacy correlation with Type 2 inflammatory biomarkers (e.g., blood eosinophil levels) provides a scientific basis for precision treatment strategies, advancing the field toward personalized management of allergic rhinitis.

### 3.3. Anti-IL5

At present, no biologics targeting IL-5/IL-5R have been subjected to specific investigation for the treatment of allergic rhinitis. Nevertheless, data from a trial of mepolizumab for the treatment of severe asthma in conjunction with a history of upper airway disease showed that Mepolizumab was associated with improved quality-of-life assessment results in patients with severe asthma and upper airway disease, including allergic rhinitis [56]. In animal testing, a study by Asakura et al. demonstrated that an anti-IL-5 monoclonal antibody suppressed early symptoms and late eosinophilia in allergic mice [57]. In a study by Saito et al., IL-5-deficient mice (IL-5(-/-)), generated via gene knockout) were compared with wild-type mice in an experimental allergic rhinitis model. The authors found that the onset of allergic rhinitis symptoms was delayed in IL-5(-/-) mice [58].

### 3.4. Anti-Thymic Stromal Lymphopoietin (TSLP)

#### Tezepelumab

Tezepelumab is a humanized monoclonal antibody administered subcutaneously, which blocks the effects of TSLP. In a randomized, double-blind, parallel-controlled trial involving 121 patients with cat allergies, a combination treatment of tezepelumab with SCIT significantly reduced TNSS induced by nasal allergen challenge (NAC) at week 52 compared with SCIT alone [59]. At week 104, there was no difference in the primary endpoint (TNSS AUC 0–1 h) between the two groups. Although TNSS peaked 0–1 h, the combination therapy group had a greater reduction. In conclusion, Tezepelumab can enhance the efficacy of SCIT and may improve patient tolerance in the 1-year treatment period [59].

### 3.5. Anti-IL33

IL-33, a pivotal alarmin cytokine within the IL-1 family, is swiftly released into the bloodstream upon bodily damage. It binds to the ST2 receptor, activating the MyD88-dependent signaling pathway. This activation leads to the stimulation of transcription factors like NF-κB and MAPK, which in turn trigger a type 2 immune response. Consequently, IL-33 plays a central role in both allergic and non-allergic inflammations [60].

In the realm of allergic rhinitis (AR) research, the anti-IL-33 therapy has demonstrated remarkable efficacy in AR animal models. In a mouse model of allergic rhinitis, treatment with anti-IL-33 significantly reduced nose scratching events, brought about an apparent improvement in skin exfoliation symptoms, and diminished the degree of eosinophil infiltration. Moreover, after intraperitoneal injection and intranasal ovalbumin challenge, both total serum IgE and ovalbumin-specific IgE levels were notably reduced, providing robust experimental evidence for the anti-IL-33 treatment of AR [61].

Etokimab, a humanized immunoglobulin subtype G1/κ monoclonal antibody, has been confirmed through in vitro studies to bind to human IL-33 with high affinity, effectively inhibiting its activity. In the treatment of allergic diseases, it has achieved remarkable results [62,63,64]. In a multi-center, randomized, double-blind, placebo-controlled phase 2a clinical trial involving adult peanut-allergic patients, etokimab not only enhanced antigen tolerance but also significantly decreased atopy-related adverse events [63]. Furthermore, in a mouse model of allergic asthma, the combined treatment of etokimab and anti-Siglec-F exhibited a significant synergistic effect. Through an additive mechanism, it effectively curbed eosinophil infiltration, further highlighting its potential in anti-allergic treatment [64].

Despite the current lack of research on using etokimab for AR treatment, considering the central role of IL-33 in the pathogenesis of allergic diseases and the significant efficacy of anti-IL-33 biologics like etokimab in various allergic conditions, such monoclonal antibodies hold great potential for application in AR treatment. They are worthy of further in-depth exploration and systematic research by scientific researchers.

## 4. Selection of Biologic Agents

Current clinical trials investigating biological agents for allergic rhinitis (AR) have primarily focused on dupilumab, omalizumab, and CM310 (detailed characteristics summarized in Table 2). Emerging evidence suggests distinct differences among these biologics regarding efficacy profiles and target populations. Based on these clinical findings, this paper provides a tentative evaluation of their respective positions in AR management.

### 4.1. Omalizumab

#### 4.1.1. Seasonal Allergic Rhinitis

Clinical studies highlight the substantial clinical utility of omalizumab in IgE-mediated SAR. Data from randomized controlled trials [65] show that a single 300 mg dose administered two weeks before pollen season onset achieves superior efficacy compared with conventional therapies, with protective effects lasting 2–4 weeks after the season ends. This proactive intervention establishes a novel framework for SAR management.

Dose-response analyses indicate the 300 mg regimen induces significantly greater improvement in nasal symptoms than the 150 mg dose, potentially mediated through dose-dependent reduction of serum free IgE levels [65]. The inverse correlation between serum free IgE and clinical efficacy [7,65] supports the clinical rationale for weight- and IgE-based individualized dosing, as validated in multicenter studies by Chervinsky et al. [66] and Vignola et al. [67].

#### 4.1.2. Perennial Allergic Rhinitis

Omalizumab remains the only biologic with completed clinical trials in pure PAR patients. After 16-week treatment, mean daily nasal symptom severity scores were significantly lower versus placebo (*p* < 0.001) [66]. Post hoc analyses revealed significant symptom reduction (*p* = 0.012, *p* = 0.022) even in subgroup refractory to prior immunotherapy or intranasal corticosteroids [66]. For PAR patients with comorbid asthma, omalizumab reduces asthma exacerbation risk by 38% (*p* = 0.02) while alleviating rhinitis symptoms [67].

### 4.2. Dupilumab

Dupilumab demonstrates unique advantages in PAR patients with co-existing type 2 inflammatory comorbidities. The Weinstein et al. trial (n = 1982) confirmed that 300 mg q2w dosing concurrently improves rhinitis symptoms (SNOT-22 reduction: 5.98 points, *p* = 0.009) and asthma control (FEV1 improvement: 0.18 L, *p* < 0.01) [49]. Its efficacy correlates strongly with type 2 inflammatory biomarkers (elevated fractional exhaled nitric oxide [FeNO], blood eosinophilia) [68], suggesting particular suitability for PAR patients with definitive type 2 inflammation. However, evidence for isolated allergic rhinitis remains limited.

### 4.3. CM310

Approved by China’s National Medical Products Administration (NMPA) on 7 February 2025, CM310 is the first global IL-4Rα antagonist approved for SAR. A q2w subcutaneous regimen provides sustained improvement in nasal/ocular symptoms and quality of life in moderate-to-severe SAR [54]. Notably, it demonstrates exceptional efficacy in patients with baseline blood eosinophils ≥ 300 cells/μL [55], similar to dupilumab, positioning it as a preferred option for SAR patients with pronounced type 2 inflammation.

## 5. Direction of Clinical Application

### 5.1. Refractory Allergic Rhinitis

Biologics have shown efficacy in treating refractory AR unresponsive to conventional pharmacotherapy. In a meta-analysis of 11 clinical trials investigating omalizumab for poorly controlled AR, Sophia et al. observed that patients with uncontrolled AR and sinusitis experienced symptomatic relief, reduced need for rescue medication, and improved quality of life with omalizumab treatment, showing a statistically significant correlation [69].

### 5.2. Seasonal Allergic Rhinitis

Most clinical trials investigating biologics for allergic rhinitis have involved patients with seasonal AR and have demonstrated promising results. Sophia et al. reviewed 12 omalizumab clinical trials for AR up to 2020. Of these, 11 studies focused on seasonal AR, with only one addressing moderate-to-severe persistent AR [66,70].

### 5.3. Combination Therapy

Combining biologics with existing therapies, such as first-line medications or immunosuppressive agents, is likely to enhance treatment efficacy and provide symptom relief. A meta-analysis of biologics combined with allergen immunotherapy (AIT) found that patients receiving omalizumab and AIT achieved target maintenance doses more frequently and had fewer systemic adverse events compared to those on AIT alone [71]. Additionally, a double-blind, placebo-controlled trial by Kopp et al. involving 140 patients with seasonal AR and comorbid seasonal asthma showed that adding omalizumab to SCIT significantly reduced daily symptom scores and modestly improved quality of life compared to SCIT alone [72].

## 6. Other Considerations

### 6.1. Long-Term Efficacy and Safety Studies

Most studies on monoclonal antibodies for allergic rhinitis have focused on short-term outcomes, with limited research on long-term efficacy. A retrospective observational study of 11 patients who received a 36-month course of omalizumab injections for severe asthma with persistent allergic rhinitis showed a sustained decrease in VAS scores, indicating prolonged efficacy [53]. However, the small sample size limits the generalizability of these findings. Adverse effects, such as allergic reactions, pregnancy-related risks, and eosinophilia, also require close monitoring [73]. For example, the Novartis safety report showed that 0.2% of 57,300 patients treated with omalizumab from 2003 to 2006 experienced anaphylactic reactions [74]. In placebo-controlled trials of berlinumab, hypersensitivity reactions occurred in 1–3% of patients, as reported in references [75,76]. The WHO Safety Reporting Database also recorded 36 cases of pregnancy-related adverse effects with dupilumab, with 58.3% of these being spontaneous abortions (OR 0.57 [95% CI 0.37–0.88]), though a direct causative link remains unproven [77]. The most commonly reported adverse reaction associated with dupilumab is conjunctivitis. However, this is typically a mild and non-serious condition that can be managed conservatively without necessitating treatment cessation [39,78]. Future studies must prioritize investigating the long-term effects of biologics and their potential adverse effects to guarantee the safety and efficacy of these agents in clinical settings.

### 6.2. Economic Cost

The high costs of biologic therapies, including anti-IgE and anti-IL-4/IL-13 agents, pose economic challenges relative to existing pharmacologic and surgical treatments. Evaluating these therapies from a pharmacoeconomic perspective is essential to assess their value and accessibility for patients. Biologics that prove effective in clinical trials should be examined to determine optimal cost-effectiveness, potentially creating more accessible options for patients with severe or treatment-resistant AR [79].

## 7. Conclusions

With the rapid development of precision medicine, biologics provide new treatment options for AR patients with different conditions and needs. Based on the immune pathogenesis, their targeted treatment features endow biologics with great potential in relieving patients’ symptoms and improving their quality of life. Although biologics are still in the research stage, with the continuous advancement of medical technology and in-depth research, biologics are expected to become a crucial treatment method for AR, providing patients with more effective treatment alternatives.

This paper tentatively proposes a multidimensional strategy for selecting biologic agents in allergic rhinitis (AR) based on key clinical trials, integrating serum IgE levels, disease phenotypes (seasonal/persistent), and comorbid conditions: omalizumab, which requires IgE-level individualized dosing, is suitable for patients with elevated IgE, while CM310 and dupilumab target type 2 inflammatory biomarkers (such as blood eosinophils and FeNO) with less dependence on IgE levels. For disease phenotypes, seasonal AR prioritizes omalizumab (for prophylactic intervention 2–4 weeks before pollen season) and CM310 (for moderate-to-severe type 2 inflammatory cases), whereas persistent AR currently relies solely on omalizumab supported by Phase III data—dupilumab is indicated in persistent AR only when combined with type 2 comorbidities like asthma or atopic dermatitis. In terms of comorbid conditions, dupilumab addresses multi-organ inflammation through targeting the type 2 pathway, making it optimal for patients with concurrent asthma or atopic dermatitis. This strategy systematically aligns omalizumab, dupilumab, and CM310 with patients based on their IgE status, disease phenotype, and comorbid profiles, offering a precision-medicine approach rooted in clinical evidence and mechanistic insights.

However, in the future, it is necessary to further strengthen the research on the long -term efficacy and safety of biologics. This involves increasing the sample size, thoroughly exploring potential adverse reactions, assessing cost-effectiveness from a pharmacoeconomic perspective, optimizing treatment regimens, and enhancing cost-performance. These efforts aim to promote the widespread application of biologics in the clinical treatment of AR and bring a safer, more effective, and more cost-efficient treatment experience to a large number of AR patients.

## Figures and Tables

**Table 1:** Biological agents targeting type 2 immune response [41,42].

Target	Name of Drug	Company	Indications	For AR R&D Status
IgE	Omalizumab	Novartis Pharmaceuticals	Allergic asthma, chronic sinusitis and nasal polyps, chronic idiopathic urticaria, and chronic spontaneous urticaria	Approved in Japan for the treatment of severe seasonal rhinitis [29]
IgE	LP-003	Tianchen Biology	Allergic rhinitis and chronic spontaneous urticaria	Phase III clinical trial in China
IL-4/IL-13	Dupilumab	Sanofi	Eosinophilic asthma, atopic dermatitis, chronic sinusitis, and nasal polyps	Experimental phase
IL-4R	CM310	Keymed Biosciences	Allergic rhinitis	Approved in China for the treatment of severe seasonal rhinitis
IL-4R	QX005N	Qyuns	Phase III clinical trial of prurigo nodosum, Phase III clinical trial for atopic dermatitis, and Phase II clinical trial of chronic sinusitis with nasal polyps	Not yet
IL-4R	TQH2722	Chiatai Tianqing	Phase II clinical trial for atopic dermatitis and Phase II clinical trial of chronic sinusitis with nasal polyps	Not yet
IL-4R	GR1802	Genrixbio	Phase III clinical trial for moderate to severe atopic dermatitis, Phase II multicenter clinical trial of chronic sinusitis with nasal polyps, b10 > Phase II clinical trial for seasonal allergic rhinitis, and a Phase II multicenter clinical trial in chronic spontaneous urticaria	Phase II clinical trial in China
IL-4R	CBP-201	Conneder	Phase III clinical trial for moderate to severe atopic dermatitis and Phase III clinical study of asthma	Not yet
IL-5	Mepolizumab	GlaxoSmithKline	severe asthma (eosinophilic phenotype), eosinophilic granulomatosis and polyangiitis (EGPA) in adults, and eosinophilic syndrome (HES) in children 12 years of age and older	Experimental phase
IL-5	Depemokimab	GlaxoSmithKline	Phase III clinical trial for hypereosinophilic syndrome and Phase III clinical trial for chronic rhinosinusitis with nasal polyps (CRSwNP).	Experimental phase
IL-5	Benralizumab	AstraZeneca	Severe eosinophilic asthma	Not yet
IL-5	BAT2606	Bio-Thera	Chronic sinusitis with nasal polyps	Not yet
TSLP	Tezepelumab;	AstraZeneca	Severe chronic sinusitis with nasal polyps Phase III study, Phase III study of severe asthma, and eosinophilic esophagitis Phase III study	Not yet
TSLP	CM326;	Connaught	Phase II clinical study of moderate to severe asthma, Phase II clinical study of moderate to severe atopic dermatitis, and Phase II clinical study of chronic sinusitis with nasal polyps	Not yet
TSLP	TQC2731	Chia Tai Tianqing	Phase II clinical trial of severe asthma and Phase II clinical trial of chronic sinusitis with nasal polyps	Not yet
TSLP	SHR-1905	Hengrui	Phase II clinical trial for asthma and Phase II clinical trial of chronic sinusitis with nasal polyps	Not yet
IL-25	XKH001	Xinkanghe	Phase I study of moderate to severe asthma and single-arm Phase Ic clinical trial for allergic asthma	Not yet
IL-33	Etokimab/ANB020	AstraZeneca	Phase IIa clinical trial of chronic sinusitis with nasal polyps and Phase IIa clinical trial of peanut allergy	Not yet

**Table 2:** Summary of key clinical trials for biologic agents in allergic rhinitis.

Clinical Trials	Inclusion Criteria	Dosage Regimens	Observation Period	Efficacy Evaluations	Baseline IgE	Baseline Scores	Efficacy Outcomes
**Omalizumab**							
Casale et al. [65]	Aged 12–75, with SAR ≥ 2 years, IgE 30–700 IU/mL.	IgE > 150: 50/150/300 mg q3W for 4 times; IgE ≤ 150: same doses q4W for 3 times.	12 weeks.	Daily nasal symptom severity score (0–3); RQLQ; rescue medication;	>150: 305.4 ± 138 IU/mL ≤150: 79.0 ± 34.0 IU/mL	Nasal: 0.71 ± 0.59; RQLQ: 1.53 ± 1.16	300 mg group showed significant symptom improvement (Δ−0.36, *p* = 0.001) with clear dose-response relationship
Zhang et al. [7]	Aged 18–60, SAR ≥ 2 years, sIgE ≥ 0.7 kUA/L	Single 300 mg injection 2 weeks before pollen season	6 weeks	CSMS-nose; CSMS-eye; TNSS/TESS; MS;	381.24 ± 85.32 kU/L	TNSS: 1.44 ± 0.24; TESS: 0.38 ± 0.12; MS: 0.44 ± 0.13; CSMS-nose: 0.68 ± 0.16; CSMS-eyes: 0.63 ± 0.16; RQLQ: 5.65 ± 0.89;	CSMS-nose reduced by 40% (*p* < 0.001); Better ocular symptom improvement (*p* = 0.004)
Chervinsk et al. [66]	Aged 12–75 with PAR, IgE 30–700 IU/mL	≥0.016 mg/kg/IgE q4W	16 weeks	Daily nasal symptom severity score; RQLQ(0–6); rescue medication	149 IU/mL	Nasal: 1.7 (0–3); RQLQ: 3.08 (0–6)	Symptom control rate 69% vs. 49% in controls (*p* < 0.001)
Vignola et al. [67]	Aged 12–74 with moderate-severe PAR ≥ 2 years; Allergic asthma ≥1 year;	≥0.016 mg/kg/IgE (IU/mL) q4W	28 weeks	Asthma exacerbation rate; QoL improvement; FEV1 change; symptom scores	30–1300 IU/mL	AQLQ: 4.0 ± 0.81; RQLQ: 3.8 ± 0.87	Exacerbations ↓32% (*p* = 0.02); QoL improvement ↑17.1% (*p* < 0.001)
**CM310**							
Zhang et al. [55]	Aged 18–65 with moderate-severe SAR ≥ 2 years	600–300 mg qW/q2W; 4-week treatment	12 weeks	rTNSS; rTOSS; RQLQ	-	rTNSS (0–12): 8.7 ± 1.9; rTOSS: 6.0 ± 2; RQLQ: 4.0 ± 1.1	Improvement trend but not significant (*p* = 0.065); notably improved nasal/ocular symptoms in eosinophil-high (≥300/μL) subgroup (rTNSS Δ−37.2% vs. −28.5%, *p* = 0.032).
Zhang et al. [54]	Aged 18–65 with moderate-severe SAR ≥ 2 years	300 mg q2W (loading 600 mg); 4-week treatment	8 weeks	rTNSS; rTOSS; RQLQ	-	rTNSS:9.2 ± 1.4; rTOSS: 6.2 ± 1.7; RQLQ:4.2 ± 0.9 Blood eosinophils 540/μL	2 weeks: the mean change from baseline in the daily rTNSS of the stapokibart group was superior to that of the placebo group (−1.3, 95% CI: −2.0 to −0.6, *p* = 0.0008); 4 weeks: (−1.7, 95% CI: −2.5 to −0.8, *p* = 0.0002).
**Dupilumab**							
Weinstein et al. [49]	Persistent asthma + PAR, sIgE ≥ 0.35 kU/L	200/300 mg q2W	24 weeks	SNOT-22 total score and AR-related items (postnasal drip, congestion, rhinorrhea, and sneezing)	-	SNOT-22: 15.27–19.30; blood eosinophils ≥300/μL: 42%	300 mg group: SNOT-22 ↓5.98 (95%CI: −10.45 to −1.51, *p* = 0.009); congestion improvement −0.60 (*p* < 0.01); rhinorrhea improvement −0.67 (*p* < 0.01)
Busse et al. [68]	Moderate-severe asthma + PAR, sIgE ≥ 0.35 kU/L	200 mg q2W (loading 400 mg); 300 mg q2W (loading 600 mg)	52 weeks	Annualized severe asthma exacerbation rate; FEV1 change; ACQ-5; RQLQ;	272–321 IU/mL	RQLQ: 1.87–2.00; ACQ-5: 2.68–2.79	Severe exacerbation rate ↓34.6% (*p* < 0.05); FEV1 ↑0.18 L (*p* < 0.01); better efficacy in blood eosinophils ≥ 300/μL

Abbreviations: FEV1, forced expiratory volume in 1; SAR, seasonal allergic rhinitis; PAR, perennial allergic rhinitis; RQLQ, Rhinoconjunctivitis Quality of Life Questionnaire; CSMS, Combined Symptom and Medication Score; rTNSS/rTOSS, Reflective Total Nasal/Ocular Symptom Score; TNSS/TESS, Total Nasal/Ocular Symptom Scores; AQLQ, Asthma Quality of Life Questionnaire; SNOT-22, 22-item Sinonasal Outcome Test; ACQ-5, Asthma Control Questionnaire (5 items); MS, medication score; Δ, indicates the change in value; ↓/↑, denotes a decrease/increase.

## Data Availability

Not applicable.
